# Sodium in the dermis colocates to glycosaminoglycan scaffold, with diminishment in type 2 diabetes mellitus

**DOI:** 10.1172/jci.insight.145470

**Published:** 2021-06-22

**Authors:** Petra Hanson, Christopher J. Philp, Harpal S. Randeva, Sean James, J. Paul O’Hare, Thomas Meersmann, Galina E. Pavlovskaya, Thomas M. Barber

**Affiliations:** 1 Warwick Medical School, University of Warwick, Coventry, United Kingdom.; 2 Warwickshire Institute for the Study of Diabetes Endocrinology and Metabolism, University Hospitals Coventry and Warwickshire (UHCW), Clifford Bridge Road, Coventry, United Kingdom.; 3 Sir Peter Mansfield Imaging Centre (SPMIC), School of Medicine, and; 4 Nottingham NIHR Biomedical Research Centre, University of Nottingham, Nottingham, United Kingdom.

**Keywords:** Endocrinology, Diabetes, Skin

## Abstract

BACKGROUND. Dietary sodium intake mismatches urinary sodium excretion over prolonged periods. Our aims were to localize and quantify electrostatically bound sodium within human skin using triple-quantum–filtered (TQF) protocols for MRI and magnetic resonance spectroscopy (MRS) and to explore dermal sodium in type 2 diabetes mellitus (T2D).

METHODS. We recruited adult participants with T2D (*n =* 9) and euglycemic participants with no history of diabetes mellitus (*n =* 8). All had undergone lower limb amputations or abdominal skin reduction surgery for clinical purposes. We used 20 μm in-plane resolution ^1^H MRI to visualize anatomical skin regions ex vivo from skin biopsies taken intraoperatively, ^23^Na TQF MRI/MRS to explore distribution and quantification of freely dissolved and bound sodium, and inductively coupled plasma mass spectrometry to quantify sodium in selected skin samples.

RESULTS. Human dermis has a preponderance (>90%) of bound sodium that colocalizes with the glycosaminoglycan (GAG) scaffold. Bound and free sodium have similar anatomical locations. T2D associates with a severely reduced dermal bound sodium capacity.

CONCLUSION. We provide the first evidence to our knowledge for high levels of bound sodium within human dermis, colocating to the GAG scaffold, consistent with a dermal “third space repository” for sodium. T2D associates with diminished dermal electrostatic binding capacity for sodium.

## Introduction

Established dogma adopted nearly 40 years ago (1) states that urinary sodium output matches dietary sodium intake in normal human physiology. This traditional model of sodium homeostasis locates sodium ions primarily within the extracellular fluid (ECF) compartment, which — together with the intracellular fluid (ICF) compartment — forms the traditional “2-space” model for sodium homeostasis. Furthermore, unlike other essential minerals and nutrients (such as calcium, iron, fatty acids, or glucose), no storage facility exists for excessive sodium ions, which are simply excreted in the urine.

Recently, our understanding of human sodium homeostasis has been transformed by insights from data that have emerged from the Mars program (led collaboratively by the National Aeronautics and Space Administration [NASA] and scientists from the European Union [EU] and Russia), conducted as part of preparation for the prolonged zero-gravity of flights to Mars (2). Due to potential concerns regarding electrolyte imbalances that may ensue, careful sodium balance studies, performed as part of this program on young healthy cosmonauts during studies in sealed metabolic units (2, 3), included prolonged studies (over many days and weeks) on urinary sodium excretion in response to diets that contain variable (but precisely measured) sodium contents. For the first time to our knowledge, data from these studies demonstrate, in humans, the existence of ultra-long (including infradian) rhythmic variations in urinary sodium excretion (4). Importantly, these rhythms occurred despite maintenance of a constant dietary sodium intake (3, 5). In addition to there being an apparent mismatch between dietary sodium intake and urinary sodium excretion over many days and weeks, another observation was that, for those cosmonauts on a high-sodium diet, there was apparent retention of sodium without associated weight gain (3). This observation is important, as it challenges established dogma that states that, in normal physiology, retention of sodium always associates commensurately with retained water (through osmosis), thereby also influencing body weight.

Despite inherent difficulties of imaging sodium ions (6, 7), recent data stemming from use of ^23^Na 3 Tesla (3T) MRI reveal a preponderance of sodium signals from the human skin (8, 9). These human-based studies corroborated data from prior rodent-based studies (10). Further preliminary data in human chronic kidney disease (CKD) suggest that sodium content within the skin closely associates with left ventricular mass and systolic blood pressure (9, 11). It has even been suggested that the location of sodium within the skin may strengthen the antimicrobial barrier function of skin and boost macrophage-driven host defence (12).

While ^23^Na MRI methods have been important in numerous attempts to quantify the levels of sodium in human skin noninvasively (13–16), implementation of this in vivo imaging methodology in the clinical arena has yet to occur. Although elevations in skin sodium concentrations have been reported in patients with CKD and hypertension (17 18), data from such studies were obtained using whole body scanners with reported sodium levels ≤ 30 mM for various pathologies (19–21). Unfortunately, this threshold is well below that typical of ECF sodium concentration (up to 130 mM; ref. 22).

Our aim was to generate data to provide the first accurate anatomical localization and quantification of free and electrostatically bound sodium within human skin ex vivo. A further aim was to develop novel insights into the dynamics of sodium within human skin, and the influence of type 2 diabetes mellitus (T2D).

To enable ^23^Na MRI and ^23^Na magnetic resonance spectroscopy (MRS) protocols for these aims, we implemented the following steps. (a) Biopsy skin samples were used for microimaging at 9.4 Tesla magnetic field strength, to enable a much higher spatial resolution than that typically achievable using whole body MRI. Furthermore, the ex vivo conditions facilitated protocol development due to reduced time and regulatory constraints. (b) The skin specimens were vacuum packed for sample preservation without chemical treatment that would otherwise have affected sodium dynamics and concentration in the tissue. (c) We utilized multiple quantum filtered (MQF) ^23^Na MRI to discern the localization of the bound ionic sodium from the free (dissolved) one in the skin. (d) The relative quantification between the 2 types of sodium states (bound and free) was obtained using 2-dimensional triple quantum time-proportional phase incrementation (TQ-TPPI) ^23^Na MRS.

### Concepts of TQ tagging of bound sodium.

**Figure 1:**
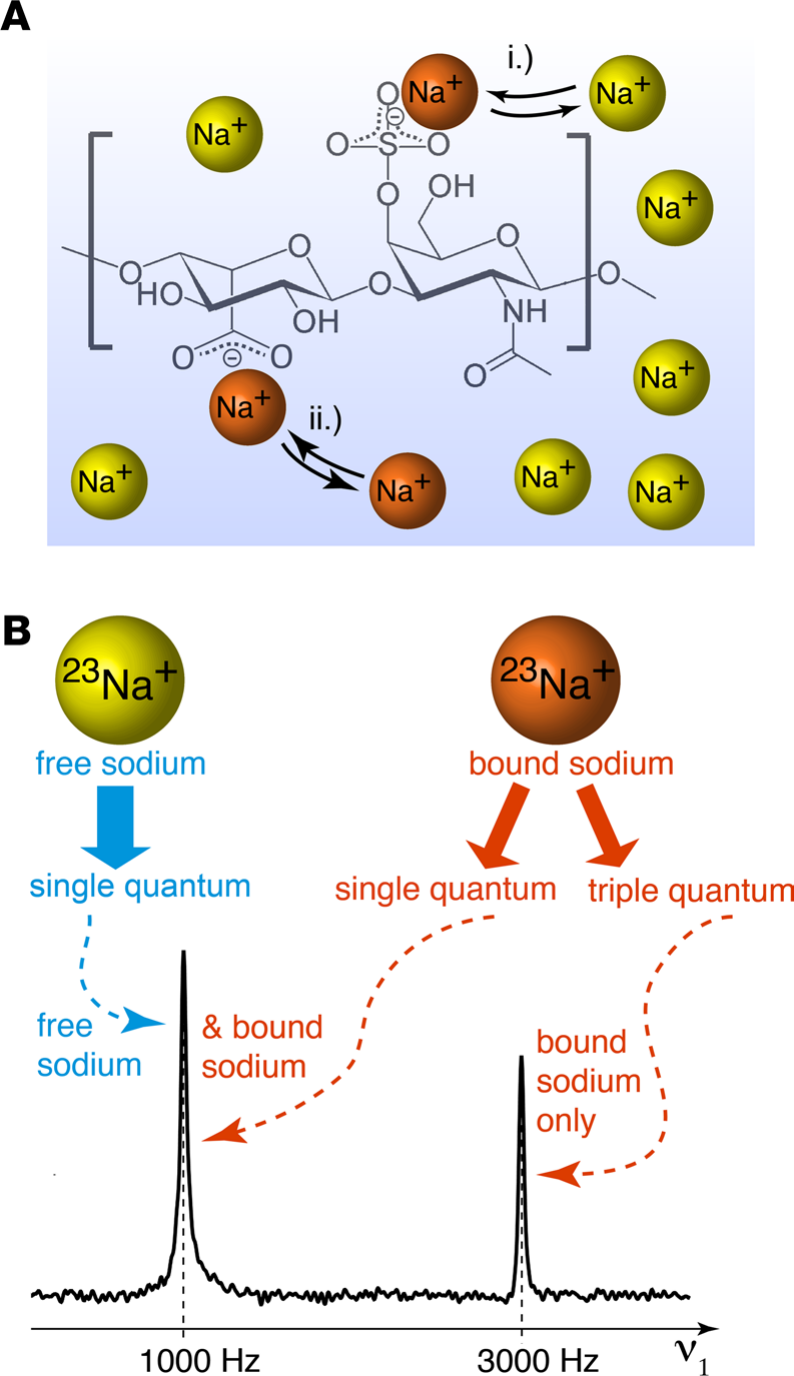
Concept of identification of bound-state sodium ions through TQF ^23^Na MRI and TQ-TPPI ^23^Na MR Spectroscopy. (**A**) Possible interactions of sodium ions (^23^Na^+^) with electro-negative sites of a glycosaminoglycan (GAG), such as dermatan sulphate. Yellow indicates dissolved (i.e., free) sodium ions; orange indicates ^23^Na^+^ in a bound or temporary bound state with the macromolecule. (**B**) Analogous to clinical and preclinical ^1^H MRI, ^23^Na MRI generates and detects single quantum coherence. However, the physical property of the ^23^Na nuclei also enables temporary generation of a TQ coherence through specific MR protocols if the sodium is bound to a slow motional molecular environment. Both, TQ-TPPI spectroscopy (shown here) and TQF MRI utilize the different properties of single quantum (SQ) and triple quantum (TQ) coherence for the differentiation between slowly moving bound sodium and rapidly moving free sodium ions.

Sodium in Figure 1A exists either in the form of dissolved ions (i.e., free sodium) within the ECF space where it undergoes rapid Brownian motion, or it is electrostatically bound to specific electronegative sites of macromolecules (i.e., bound sodium), in which state the ^23^Na motion is reduced substantially. At sites with slow exchange rate, there is fixation of the sodium ions for the duration of the MRI measurement (timescale of hundreds of milliseconds, indicated by small arrowheads), even when multiple exchanges occur during the measurement period, and the net ^23^Na motion may be slowed substantially compared with that of the free ions. For this study, we consider sodium that undergoes slow exchange in the ways described here as bound sodium.

The ability to distinguish between free and bound sodium in magnetic resonance (MR) measurements arises from the high nuclear spin (S = 3/2) of ^23^Na. A TQ coherence can be induced through specific MR protocols if the ^23^Na is electrostatically bound to a large molecular environment that imposes a slow-motional state for the sodium. Since it is not possible to generate TQ coherence in fast-moving free sodium ions (i.e., for ^23^Na in a fast-motion state), it is possible to use this difference in signal to “tag” specifically bound sodium. In addition, TQ-TPPI ^23^Na MRS, illustrated in Figure 1B, enables quantification of the ratio of bound to free sodium (23, 24). Similarly, insertion of a TQ filter (TQF) into sodium MRI protocols suppresses all signals except TQ-tagged ^23^Na; recorded MRI depicts bound sodium only. TQF MRI and TQ-TPPI MRS are well developed and understood (23, 25) and have been utilized previously in biomedical studies to distinguish between sodium within the ICF and ECF in cell suspensions spectroscopically (26) and visualized using TQF ^23^Na MRI in the brain (27, 28), in the muscle (29), and in the cartilage (30). Furthermore, standard gradient echo (GE) ^23^Na MRI protocols largely suppress signals arising from bound sodium due to its inherent fast T2 relaxation. Therefore, GE-based ^23^Na MRI protocols used in the clinical setting predominately reveal signals from free sodium ions, whereas TQF ^23^Na MRI localizes bound sodium.

## Results

**Figure 2:**
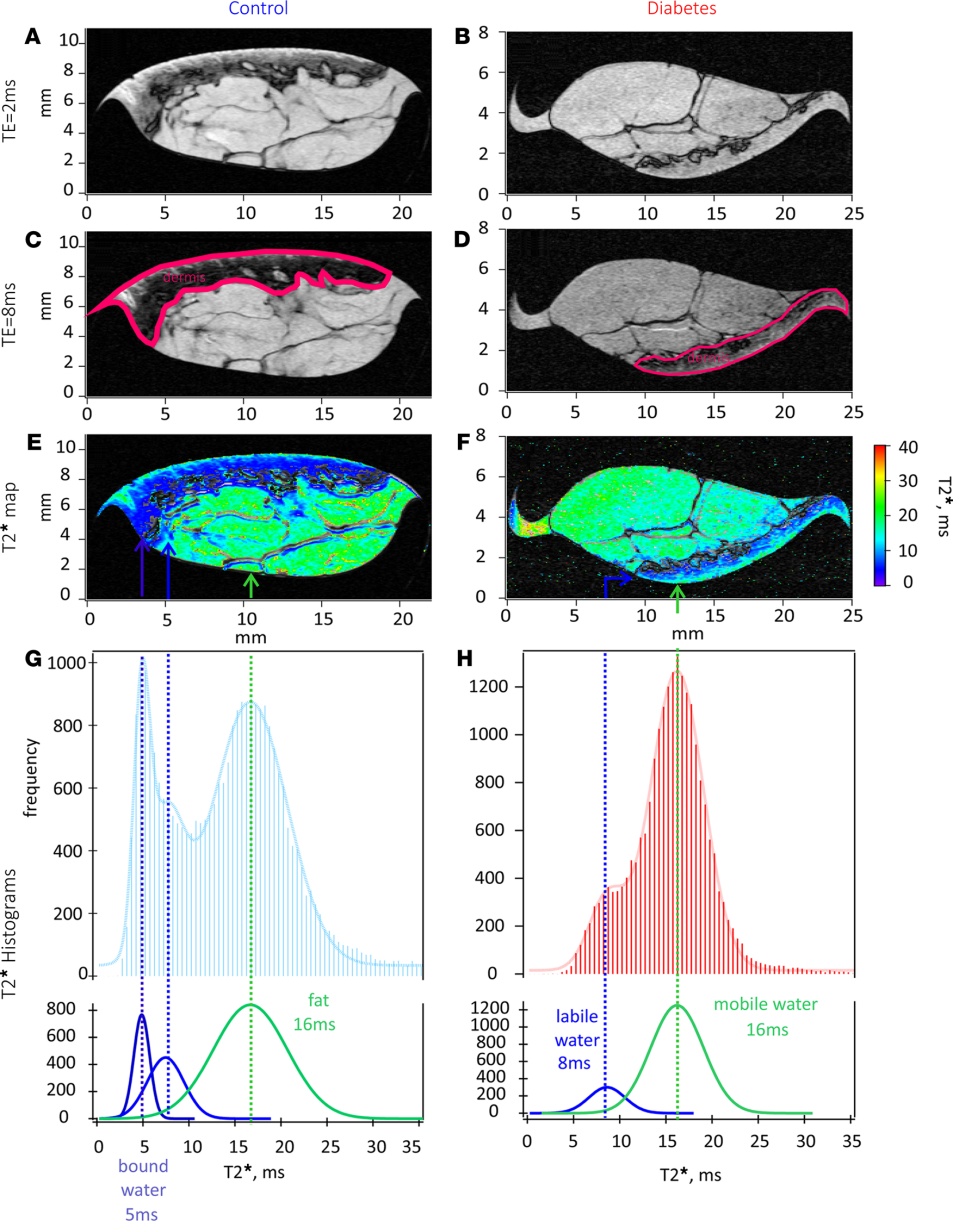
Comparison of T2* maps between control and diabetic skin biopsies for mobile and immobile (bound) water. (**A** and **B**) Anatomical compartments of human the skin using ^1^H 9.4T MRI, approaching histological length scales (20 μm) for control (NDB2) and diabetic (DB3) skin biopsies. (**C** and **D**) Segmentation of skin dermis layer in control (**C**) and diabetic (**D**) biopsies with the increase of TE to 8 ms. (**E** and **F**) T2* maps for control (**E**) and diabetic (**F**) cases overlaid with anatomical slides to highlight areas of s.c. adipose tissue (long T2* values) and aqueous phase (short T2* values). (**G** and **F**) Distributions of T2* values associated with each phase are shown in **G** for control and in **H** for the diabetic biopsy. Note the appearance of immobile or bound water at T2* = 5 ms in the control biopsy.

To discern the location for bound sodium within the skin, high in-plane resolution (up to 20 μm) ^1^H MRI was performed on selected skin biopsies, since the outcomes of high resolution ^1^H MRI were similar to each other. MRI allowed for clear identification of the major regions of the skin (epidermis, dermis, s.c. fat layer, and the microvasculature), as shown in Figure 2, A and B. As the echo time was increased in the specialized T1 weighted/T2* MRI protocol, the skin regions with decreased water mobility were further highlighted, as shown in Figure 2, C and D; the darker layer was identified as dermis in both control and diabetic skin biopsy samples. Collecting a few more echoes during this protocol allows one to convert echo images into T2* contrast images, shown in Figure 2*,* E and F. Further analysis of T2* distributions in skin biopsies from both control and diabetic cases revealed similar characteristics regarding fat and labile water compartments. However, images from the control skin biopsies also revealed the presence of extremely immobile water (with T2* of approximately 5 ms), a feature that was absent in the diabetic skin biopsies, as shown in Figure 2, G and H. The extremely immobile water observed in the control skin biopsies most likely originated from the dermis layer, as reflected by the T2* color map, over-layered with a corresponding anatomical intensity map.

**Figure 3:**
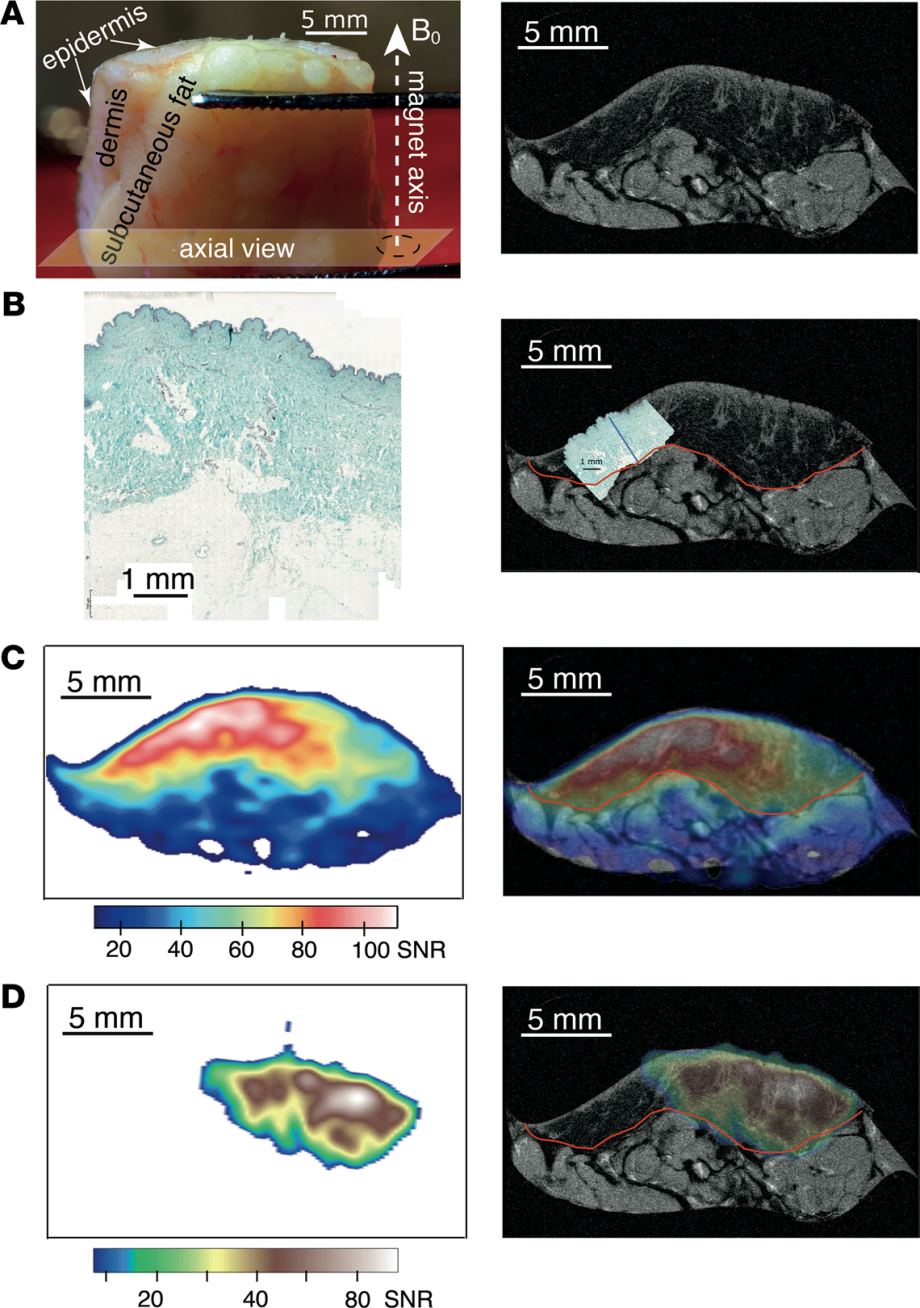
Illustration of ^23^Na MRI biopsy. (**A**) Photograph of a typical skin sample with indication of MRI axial view, left, and a high-resolution ^1^H MRI slice (11 × 11 × 100 μm^3^, axial view) showing the anatomy of the DB9 skin sample (right). (**B**) Histological GAG staining of typical skin specimen (i.e., harvested from NDB8) (left) and histology slice scaled to match the scale of the ^1^H MRI slice of DB9 to demonstrate that GAGs are localized predominantly in the dermis layer (right). The boundary of the dermis layer is indicated with red line, while blue line reflects the depth of GAG staining determined from histology. (**C**) Localization of free sodium in DB9 obtained through ^23^Na MRI (left) and its overlay with skin anatomical ^1^H MRI slice of DB9 (right). (**D**) Localization of bound sodium in DB9 obtained through TQF ^23^Na MRI (left) and its overlay with skin anatomical ^1^H MRI slice of DB9 (right). Note that free and bound sodium coregister predominantly with the dermis layer where GAGs are located. Sodium concentrations are expressed through the observed ^23^Na signal/noise ratios (SNRs).

To achieve coregistration of free and bound sodium with both skin anatomy and histology, we performed ultra–high-resolution proton scanning of a selected skin biopsy from a participant with T2D and histological glycosaminoglycan (GAG) staining of a selected skin biopsy obtained from a control participant. The resultant anatomical ^1^H MRI slice is displayed in Figure 3A, extracted from the center of the MRI multislice package with geometry axes. Figure 3B displays histological GAG staining of a skin specimen obtained from NDB8 and shows the overlay of the histology slide with ultra–high-resolution ^1^H MRI that demonstrates that stained GAGs shown in the histology slide colocalize with the dermis layer in the MR slide. Figure 3, C and D, display localization of free and bound sodium, respectively, and their overlay with ^1^H MRI skin anatomy. As one can see from Figure 3, both free and bound sodium colocalize with the dermis layer in the MRI slide, where GAGs are typically located, as determined by histological staining. We observed similar patterns for both free and bound sodium in nondiabetic skin biopsies (Supporting Information, Supplemental Figures 1–3; supplemental material available online with this article; https://doi.org/10.1172/jci.insight.145470DS1). Sodium levels were expressed in sodium signal/noise ratios (SNRs) to account for variations in the signal to noise. SNR-based approaches for quantifying sodium levels in tissues were reported before by other research groups (20, 31).

**Figure 4:**
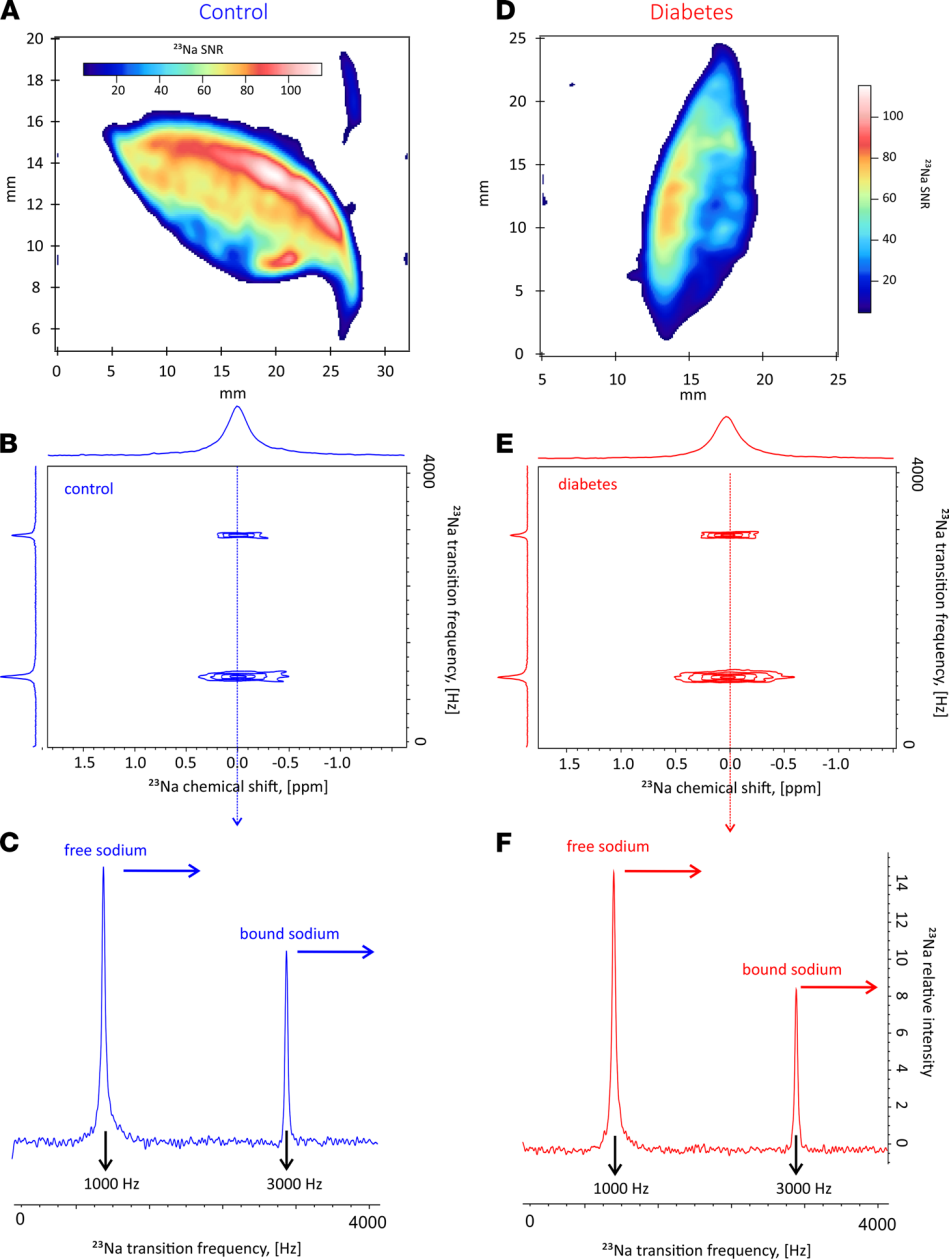
Axial localization of sodium using ^23^Na GE protocol. (**A** and **D**) The control (NDB7) (**A**) and diabetes (DB9) (**D**) skin biopsies. (**B** and **E**) Two-dimensional ^23^Na TQ-TPPI maps for control (**B**) and diabetic biopsy (**E**) using the same samples as in **A** and **D**. (**C** and **F**) Individual zero frequency traces for control (**C**) and diabetes (**F**) showing free (1000 Hz) and bound (3000 Hz) sodium signals. Note, relative to the free sodium, there is lower sodium storage in diabetes than in the control.

**Figure 5:**
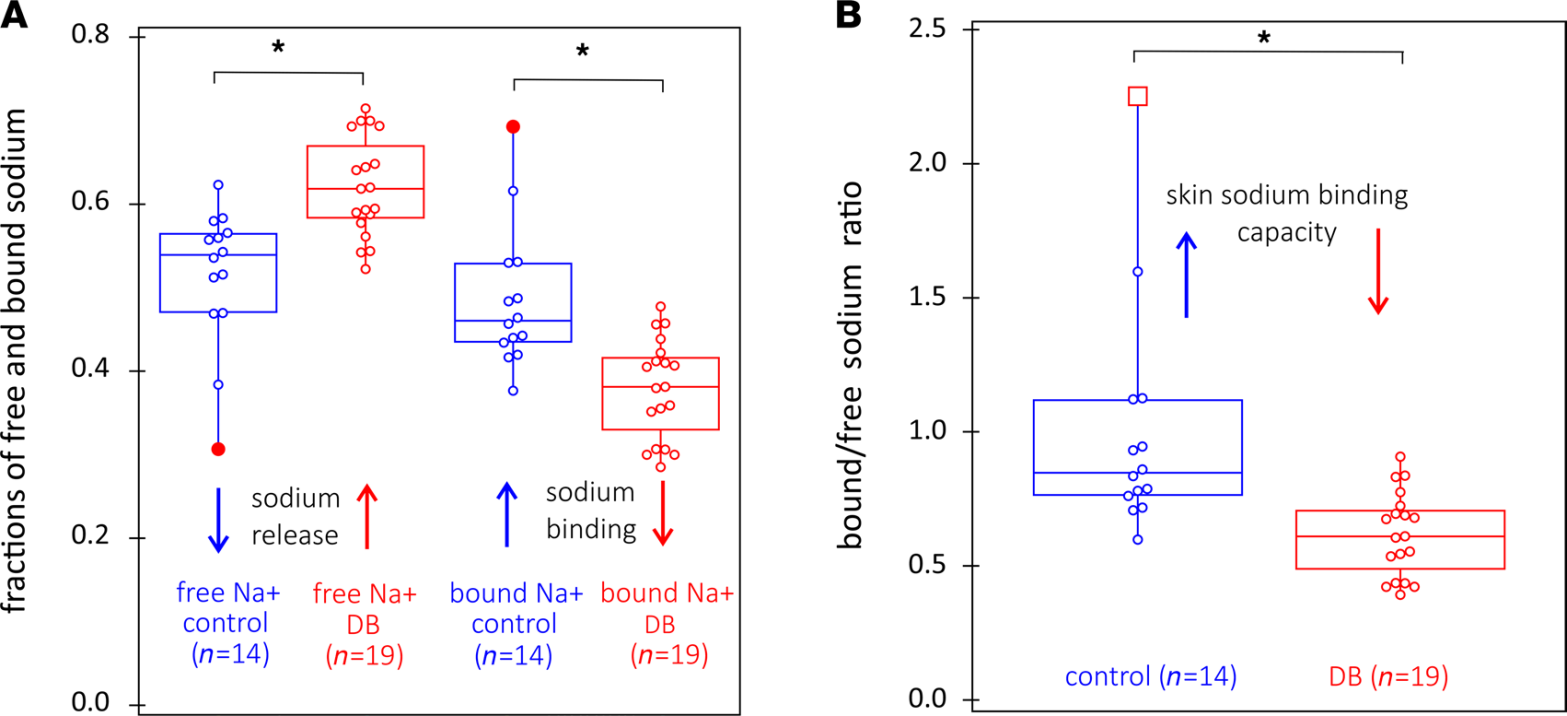
Wilcoxon signed-rank statistics of free and bound sodium fractions. Wilcoxon signed-rank statistics of free and bound sodium fractions determined from 2-dimensional ^23^Na TQ-TPPI maps, and of skin sodium binding capacity, defined as bound to free sodium ratio, for control (*n* = 14 collected from 5 patients) and diabetic (*n* = 19 collected from 9 patients) skin biopsies. (**A**) Compared with T2D, control skin samples had greater retention of free sodium release, with *P* < 0.05, while there is an increase in bound sodium in controls, with *P <* 0.05. (**B**) There is augmentation of skin sodium binding capacity for skin samples from controls versus those from patients with T2D, with *P <* 0.05, as shown in **B**.

We performed ^23^Na MRS and MRI scans in all harvested skin biopsies. We show phenotypic details of the patients who donated skin biopsy samples in Table 1. Sodium storage within each skin biopsy was further quantified by determining the fraction of sodium that would be in the bound state using 2-dimensional TQ-TPPI ^23^Na MRS (23 25), in the statistically significant pool of skin biopsies collected from patients (regardless of their T2D status). We performed sodium localization in all harvested skin biopsies prior to the application of TQ-TPPI ^23^Na MRS protocol. The images demonstrated sodium signals in similar locations, with typical examples for control (NDB7) and diabetic (DB9) skin biopsies shown in Figure 4*,* A and D, respectively. The resultant 2-dimensional sodium TQ-TPPI spectra for the skin samples imaged in Figure 4, A and D, were collected under 2 minutes and are displayed in Figure 4*,* B and E, for control and diabetic skin samples, respectively. Both 2-dimensional spectral maps were characterized by 2 signals indicative of sodium being present in both its free and bound states within each skin sample. As demonstrated from the images, about 90% of sodium recorded by the standard ^23^Na GE MRI protocol was localized in the dermis layer. Therefore, the highlighted sodium dynamics most likely originate from the dermal layer of the skin. For clarity, we further deduced the dynamics of this process through analysis of individual traces extracted from these 2-dimensional spectral maps at the zero frequency offset, as shown by dashed arrows in Figure 4, C and F. The sodium signals appearing at 3000 Hz in these individual traces reflected the amount of bound sodium, while the sodium signals appearing at 1000 Hz were indicative of sodium in its free state. As shown by the horizontal arrows, there is a diminished bound sodium content in T2D compared with controls. To test this hypothesis, fractions of bound and free sodium were determined from the areas of sodium peaks at 3000 Hz and 1000 Hz, respectively, by fitting each line shape to a Lorentzian function (32). The ratio of bound to free sodium was determined for most of the biopsies as shown in Table 1, and statistics of all data are displayed in Figure 5.

As observed from Figure 5A, there is diminishment of the free sodium fraction, or “sodium release” in control skin biopsies. Conversely, there is diminishment of the bound sodium fraction in the diabetic skin biopsies. Furthermore, the bound/free sodium ratio (shown in Figure 5B), is clearly higher for the control versus the diabetic skin biopsies, consistent with a diminished electrostatic binding capacity for sodium within the skin dermis layer from patients with T2D, compared with that from control nondiabetic patients. This difference in electrostatic binding capacity for dermal sodium between T2D and controls was independent of the anatomical location of the skin biopsy, as skin biopsy data from both abdominal and lower limb regions contributed to the statistical analyses displayed in Figure 5.

Through conversion of sodium image intensity into sodium concentration (assuming almost complete bound sodium signal loss and almost full signal recovery from the free sodium when GE ^23^Na MRI is used for image acquisition, and, conversely, assuming almost complete signal recovery of the bound sodium and full suppression of free sodium signal with the TQF is incorporated into the MRI protocol), it was possible to obtain absolute quantification of sodium from sodium MRI images, both in its free state within the ECF and electrostatically bound within the skin dermis layer. We based this conversion on assumptions that almost complete bound sodium signal loss with almost full signal recovery from free sodium loss occurs during GE ^23^Na MRI and that almost no bound sodium signal is lost with application of the TQF ^23^Na MRI protocol, which suppresses free sodium signal completely.

**Figure 6:**
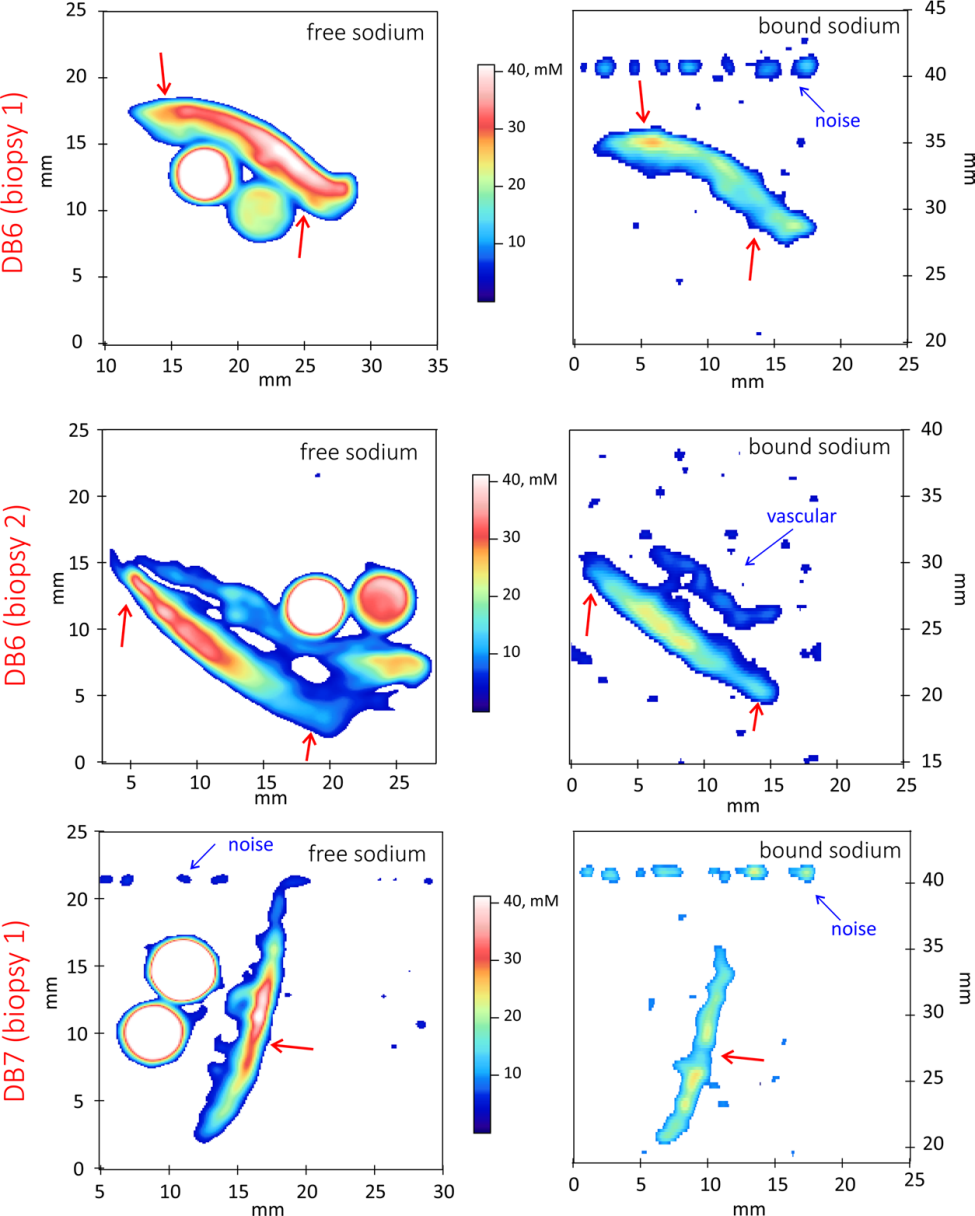
Quantification of free and bound sodium levels obtained from sodium imaging standards for 3 biopsies collected from 2 different patients. Note complementary inverse contrast converted to sodium levels in mM. Free and bound state sodium are indicated by red arrows. This implies heterogeneity of sodium deposition in the skin. Note the bound sodium in the endothelial tissue of large blood vessel shown in the right middle panel. We indicate the random noise registered at sodium frequency by blue arrows. Sodium levels are determined within ± 7 mM using saline standards.

We performed sodium imaging in 3 separate diabetic skin biopsies harvested from 2 patients (DB6 and DB7), together with known sodium standards for calibration, as shown in Figure 6. As shown in the images, maximum sodium skin concentration was determined to be 41 mM, a much lower value than 76 mM, which is a mean estimate of sodium skin concentration derived from measurements on DB6 and DB7 skin samples (Table 2). We derived the maximum sodium skin concentration from the same skin samples (DB6 and DB7) using the technique of inductively coupled plasma mass spectrometry (ICPMS, as described in Methods). Dermal sodium levels determined using in vivo MRI were reported to be < 30 mM (18, 20, 33). We attribute the higher levels of sodium obtained from our study to the finer resolution of our measurements and, hence, better segmentation of the skin dermis layer where there is predominant location of sodium, compared with those obtained from in vivo techniques. However, the sodium concentration of 41 mM in the skin dermis layer obtained in Figure 6, through comparison with known sodium standards, is still much lower than the skin sodium concentration of 76 mM, determined by ICPMS as the mean average of 2 samples extracted from 2 diabetic patients (Table 2). The fact that both sodium levels obtained from prior in vivo MRI, and free sodium levels derived from MRI of skin samples reported from our own study, are lower than the gold standard measurements most likely reflect loss of signal from bound sodium during GE ^23^Na MRI collection. As explained above, visualization of bound sodium requires incorporation of TQF into the sodium imaging protocol, and we show that the fraction of bound sodium quantified by sodium TQ-TPPI MRS (Figure 4 and Figure 5) appears to be significant. Therefore, image collection using a vendor optimized for a clinical imaging standard sodium GE protocol visualizes predominately free sodium in tissues and results in underestimation of sodium concentration within the skin as previously reported by other research groups (31).

Incorporation of the optimized maximal bound sodium image quality TQF (Supporting Information, Supplemental Figure 2) into the sodium imaging protocol (32, 34) allows one to visualize bound sodium deposition within the skin, as shown in Figure 6 (right). Using the same calibration standards as in the case above, we estimate that the maximum bound sodium is at a level of 26 mM. Adding these 2 numbers, one arrives at a value of 67 ± 7 mM for total sodium concentration using MRI saline standards (Figure 6), compared with 76 ± 5 mM as determined by ICPMS. We note the absence of sodium signals from the standards in the bound sodium images (Figure 6, right), which indicates that sodium signals originating from a liquid (or in the case of a skin biopsy, ECF) are removed as intended. Therefore, following the addition of the TQF to the imaging protocol, skin biopsy MRI indeed visualizes only bound sodium.

## Discussion

The use of vacuum-packed skin samples in the present study enabled insight into the structure of human skin at a resolution of tens of microns, approaching histological-length scales. Previous comparisons of vacuum-packed cartilage samples and their subsequent histology after MRI experiments demonstrated that the tissues remain in a viable state after being vacuum packed and refrigerated for up to 7 days (35). Since vacuum-packed materials were not exposed to any additional treatment, we believe that this method of tissue transport enabled capture of the dynamics of water distribution and sodium deposition in control and diabetic skin samples similar to that in vivo. The < 20 μm ^1^H MRI allowed for anatomical resolution of the different skin regions, and similar structural compartments were identified in vivo (36). In contrast, the T2* ^1^H MRI contrast imaging demonstrated the presence of immobile (T2* = 5 ms) water in the nondiabetic skin biopsies, localized to the layer of the dermis. This type of water was absent in the diabetic biopsies. While this result is promising and may represent a pathophysiological feature of T2D, the translation of this concept into a clinical setting is difficult. Due to its nature, the T2* ^1^H MRI contrast is more pronounced at a higher magnetic field strength (i.e., 9.4 Tesla magnetic field strength used in the present study) and diminishes with reductions in the field strength. Given the operational strength of a clinical MRI scanner being up to 3 Tesla, T2*-associated features most likely will not be observable using such scanners (37).

The quest for the identification of pathophysiological features of T2D using sodium signals has great potential for clinical translation, as the typically applied magnetic field strength only partially affects sodium T2 relaxation. We visualized sodium in both its free and bound states using sodium MRI. The usage of the TQF ^23^Na MRI protocols to detect bound sodium is well established (27, 28, 30, 32). As outlined above, sodium in fast exchange with macromolecules may transfer some TQF signals into the dissolved state of free sodium (see ii in Figure 1A), thereby potentially resulting in an overestimate of bound sodium quantity. This effect is likely present in the intracellular space and is previously used to distinguish between intra- and extracellular sodium (26).

The ratio of bound/free sodium is usually substantially lower than that reported in our study. Schepkin et al. calculated the bound sodium signal in an in vivo rat brain to be about 16% of that arising from free sodium (25), almost identical to that shown in an agarose tissue model (24). In our reported study, the signal from bound sodium in the skin dermis layer was about 90% (median value in controls), with the remaining sodium signals originating from free sodium. Therefore, our data reveal > 5-fold greater bound sodium within the skin dermis layer compared with that in other organs shown in previously published studies (25). This observation strongly suggests that, within the skin dermis layer, a high proportion of bound state sodium indeed occurs, without any attendant osmotic effects, or at least with only a minimal osmotic effect from bound sodium undergoing exchange with free sodium. We hypothesize that a substantial fraction of the bound sodium signal from the skin dermis layer, which is largely void of intracellular space, originates from stored sodium. We provide further support for this interpretation in Figure 6. Such reversal in intensity contrast for bound and free sodium would be unlikely if the TQF MRI signal is caused by rapidly exchanging sodium ions in the intra- or extracellular fluid, as in this scenario the areas of “bound” and free sodium should be identical. Further investigation of this phenomenon is, therefore, important and may provide additional valuable insight into the regional sodium deposition in the skin in health and disease. This aspect of investigation was outside the scope of the current work.

We further probed the dynamics of sodium binding or, as we hypothesize, storage using sodium MRS. Vacuum-packed samples provided a unique opportunity for this endeavor, given the absence of any additional sodium treatment introduced into the biopsied skin samples. The coregistration of proton and sodium images allowed us to visualize ^23^Na presence at the < 20 μm length scale. This confirmed that free sodium locates mostly within the dermis layer of the skin and where, according to histology, GAGs are located (Figure 3). Moreover, we localized bound-state sodium predominately within the dermis layer of the skin for both control and diabetic skin biopsies. Through analysis of both free and bound-state sodium spaces in the control and diabetic skin samples, we demonstrated that sodium release into its free state predominates within the skin samples from patients with T2D, whereas reverse sodium transfer into its bound state predominates in the skin samples from control nondiabetic patients. Our data align with previous in vivo observations suggesting that overall levels of skin sodium (probed by ^23^Na MRI) are higher in diabetes mellitus (DM) (18, 20), as the sodium MRI used in clinical practice is sensitized toward free sodium and suppresses the signal from bound sodium that requires a TQF MRI protocol to be visualized (32). Therefore, it appears that the higher sodium levels observed within the skin of patients with DM, as reported from prior ^23^Na MRI observation (18, 20), may in fact result from the detection of an increased fraction of free sodium. The ratio of bound/free sodium reflects the storage capacity for sodium within the skin. Due to the hypothesized reduction in the storage capacity for sodium within the skin dermis layer of patients with T2D, this allows for greater availability of free sodium, observed using clinical sodium MRI protocols. Importantly, our data confirm that total sodium levels in the skin dermis layer are equivalent between control and diabetic skin biopsies, as shown by ICPMS analysis in Table 2.

Recent work (38) suggested that sodium levels in skin biopsies extracted from CKD patients and controls were similar. Though we performed ICPMS only in one control sample, we demonstrated that the average sodium concentration, determined from 3 samples taken from different regions of the skin dermis layer, was 79.8 ± 5.0 mM compared with 75 ± 5.0 mM for the diabetic biopsies, again showing that total sodium levels in the skin dermis layer are similar in both cases. We hypothesize that multiple factors influence the proportion of sodium in its free and bound states. The former sodium state localizes within the interstitial fluid of the dermal extracellular matrix (ECM); we hypothesize that the latter complexes with the negatively charged sites of the GAG scaffold within the skin dermis layer. These factors include temperature and vascular and lymphatic flow.

Observations from the Mars program (including mismatches between daily sodium intake and urinary excretion of sodium, as well as mismatches between net sodium status and body weight through retained sodium) cannot be explained through our traditional physiological understanding of the “2-space” model of human sodium homeostasis, implicating the 2 compartments of ICF and ECF (1). Our reported data challenge this traditional model of human sodium regulation, at least ex vivo. A more sophisticated model of sodium homeostasis is required — one capable of providing an explanation for the mismatches between sodium intake and excretion and between net sodium status and body weight observed in the Mars program. This model of sodium homeostasis invokes the existence of a third space repository (TSR) for sodium ions, capable of storing sodium ions in a location that is both osmotically and electrostatically inert, and separate and distinct from both the ECF (16) and the ICF. A “third space” model of human sodium regulation would be entirely consistent with the observations from the Mars program. These include the storage of sodium without associated weight gain, and mismatches between dietary sodium intake and urinary sodium excretion, each of which would be permissible and indeed predictable from a “third space” model of sodium homeostasis.

From an evolutionary perspective, a “third space” model of sodium homeostasis with a TSR for sodium would be entirely logical and similar to storage repositories for other essential minerals and nutrients (such as calcium and fatty acids within the skeleton and adipose tissue, respectively). Such repositories enable potentially life-saving supplies of essential minerals and nutrients to the body in times of need — during starvation, for example. Given the central role of sodium in the maintenance of an adequate blood volume, a TSR for sodium would serve as a source of sodium for the ECF in times of acute loss of sodium, such as occurs during diarrhoeal illness or catastrophic blood loss. In such scenarios, release of arginine vasopressin (AVP) in response to movement of sodium ions from a TSR to the ECF would mitigate against loss of plasma volume through renal retention of water. This mechanism would confer substantial survival and, therefore, evolutionary advantage. Conversely, any compromise of the TSR for sodium would likely associate with a commensurate increase in free sodium within the ECF, as well as with promotion of hypertension and adverse cardiovascular sequelae.

Based upon the presented experimental evidence that bound sodium locates mostly within the skin dermis layer, we propose that, given the high negative charge of the dermal GAG scaffold, this would attract electrostatically and bind positively charged sodium ions naturally from their free state within the surrounding and contiguous ECF. In this way, the GAG scaffold within the skin dermis layer, located within the ECF itself, would function as a natural TSR for stored sodium. Bound sodium stored within the proteoglycan matrix of the GAG scaffold of the skin dermis layer could exchange with the surrounding unbound and free sodium within the ECF; however, this exchange would be limited to the ECF pools encapsulated into gaps formed by proximal GAG chains. Hence, we envision that only a proportion of bound sodium stored within the skin dermal layer GAG scaffold would participate in this exchange and that the sodium “captured” by the GAG network is best defined as stored sodium. To change the capacity for sodium storage within the skin dermal layer GAG would require remodeling of negatively charged sites. Such remodeling could result from a change in the GAG conformation due to exposure to altered calcium levels, for example (39). Alternatively, a change in the production of GAGs and their polymerization (40) could result in a change to the number of possible sodium exchange sites and their exposure to the surrounding ECF. Greater extension of the GAG scaffold into the surrounding ECF would likely increase its overall storage potential for sodium. Our data suggest that control nondiabetic skin associates with optimized storage capacity for sodium within the skin dermis layer. Conversely, we hypothesize that a possible structural change and/or reduced production of the dermal GAG in patients with T2D results in diminished storage capacity for bound sodium. It has also been proposed that the GAG complex within the endothelial layer may act as an intravascular buffer that enables transient storage of sodium (41, 42).

Unlike free sodium within the ECF and ICF, bound and stored sodium within the skin dermis layer TSR would not have osmotic effects and, therefore, would not associate with either peripheral oedema or deleterious hemodynamic effects. This would help to explain the observations from the Mars program regarding mismatched net sodium intake and body weight (from retained water through osmotic effects) (3, 40). The GAG scaffold within the skin dermis layer provides an ideal location within the ECF to act as an effective buffer for sodium overload in conditions that associate with sodium retention. Furthermore, the dermal TSR for sodium also provides an ideal location for the release of bound stored sodium to its free state into the surrounding ECF, at times of acute sodium loss and need (following diarrhoeal illness or acute blood loss from trauma, for example). At such times, we hypothesize that stored sodium ions might simply migrate from the dermal TSR into the surrounding ECF — possibly through the collapse of GAG chains within the skin dermis layer, thus decreasing the number of negatively charged sites available for sodium complexation — and by implication resulting in a diminished dermal TSR for sodium. Once released from its bound stored form, sodium would exist in the ECF in its usual free (unbound) state and, through osmotic and AVP-related effects, enable restoration of a functional ECF and plasma volume. We also hypothesize that a dermal TSR for sodium would act as a buffer to protect the surrounding ECF and plasma volume from the potentially deleterious osmotic and hemodynamic effects of excessive unbound sodium, and we hypothesize that it would provide a mechanism to accommodate large quantities of exchangeable bound sodium resulting from chronic sodium retention, as often occurs in DM (43). The dermal sodium TSR is a dynamic structure, and its dynamic amplitude may provide a useful marker of pathology, including, for example, heart failure (44).

To summarize, we provide the first evidence to our knowledge for high levels of bound sodium within human skin dermis layer, colocating to the GAG scaffold, and we provide support for the hypothesis that at least 40% of dermal sodium — that exists within a TSR — is bound and stored in the dermis. Furthermore, we also provide the first evidence to our knowledge for an association between T2D and a compromised dermal binding capacity for sodium. Our study highlights the importance of full characterization and quantification of dermal sodium using sufficiently powerful MRI techniques, for proper and accurate evaluation of bound dermal sodium. Unfortunately, application of sodium MRI techniques used in clinical practice typically lack sufficient power to enable such accurate measures of dermal sodium because signals arising from bound sodium usually remain silent. Given the technical difficulties of localizing a TSR for sodium using clinical MRI scanners, including image resolution and scan time, we propose a revision of sodium acquisition strategies for routine clinical purposes. This is especially relevant in the application of such techniques for the interpretation of skin sodium levels in patients with DM. A major challenge would be in the translation of 2-dimensional ^23^Na MRS like TQ-TPPI to in vivo clinical scanners, though this task is technically achievable. The benefit of this technological investment would be the delivery of a clinically relevant noninvasive sodium protocol that could accurately quantify bound sodium within the dermis within minutes, thereby providing insight into the capacity of the dermal TSR for sodium in patients with DM and other medical conditions. The availability of such a clinical tool would provide invaluable insight into the sodium status of patients throughout the entire realm of clinical medicine and help to inform appropriate and timely management, thereby improving patient care and saving lives. Data from such a tool would also inform future guidance on the effective management of sodium homeostasis in patients with DM and other relevant conditions.

## Methods

### Study design.

The skin samples were obtained from patients with a confirmed diagnosis of T2D (*n =* 9) and control patients with no known history of DM (*n =* 8); phenotypic details are shown in Table 1. All skin samples derived from patients with T2D came from the lower limb. For the controls, skin samples came from the abdomen (*n =* 5) and lower limb (*n =* 3). Lower-limb amputations and abdominal surgery were performed for clinical reasons. We only derived skin samples from normal skin (proximal to any area of infection or necrosis in the case of amputations), confirmed both macroscopically and with histological examination. Regarding glycemic therapies at the time of skin resection, most of the patients with T2D (*n =* 6) were on insulin therapies; one of the patients was taking a sodium glucose-like transporter 2 (SGLT2) inhibitor, metformin, and a glucagon-like peptide 1 (GLP1) agonist therapy, and one was taking a sulphonylurea and dipeptidyl peptidase 4 (DPP4) Inhibitor therapy. Regarding antihypertensive therapies in the T2D patient group at the time of skin resection, some (*n =* 3) were taking angiotensin converting enzyme (ACE) inhibitor therapies, some (*n =* 3) were taking diuretics, and one was taking an angiotensin II receptor blocker therapy. For the control group at the time of skin resection, most (*n =* 7) were not taking any therapies, and one was taking a diuretic and ACE inhibitor therapy.

Each skin sample (measuring approximately 2 × 2 × 0.5 cm^3^) was vacuum packed to preserve its natural sodium distribution, stored on ice, and delivered on ice to SPMIC, via courier within 2 hours of surgical resection. We scanned all skin samples (as described below) at 37°C, after their delivery to SPMIC. We checked the viability of vacuum-packed tissues by 1-dimensional ^23^Na MRS using “1-pulse acquire sequence” to characterize free sodium and TQF sequence for bound sodium. Prior to performing the experiments, we observed no difference in spectra between scanning upon the arrival, after 1 day of keeping the tissues at 37°C and following storage of the samples at a refrigerated (5°C) temperature for 1 week.

### General NMR and MRI.

Nuclear magnetic resonance (NMR) and MRI of all spin species reported in this study were performed on a 9.4 T Bruker Avance III Microimaging system (Bruker). We used single-channel 25 mm commercial microimaging coils (Bruker) and a dual tuned ^1^H/^23^Na microimaging coil (Bruker). We used coils tuned to the resonance frequency of 400.18 MHz for protons and 105.86 MHz for sodium for all ^1^H and ^23^Na skin sample studies. The length of the ^1^H π/2 pulse was 32 μs at 150 W, the dwell time was set to 40 μs, and the resonance offset was set at the maximum of the proton peak height associated with water phase in each experiment, or a series of consecutive experiments. Usually, we observed 2 proton peaks associated with the aqueous and the lipid phases in the 1-dimensional proton spectrum. The length of the ^23^Na π/2 hard pulse was 48 μs at 200 W, the dwell time was set to 80 μs, and the resonance offset was set at the maximum of the sodium peak height in each experiment — or a series of consecutive experiments, as we observed only 1 peak. We performed all sodium experiments at 37°C unless otherwise stated. We have provided specific experimental spectroscopic and imaging details below.

### ^1^H MRI.

Anatomical images were acquired using Paravision 6.0 (Bruker). We performed each anatomical imaging protocol using a build-in multislice GE imaging protocol with slice thickness of 200 μm. Each anatomical experiment contained up to 30 slices, to highlight possible heterogeneities in the skin biopsy structure. The images were acquired using full π/2 flip angle, with Time to Echo (TE) = 8 ms. Recycle delay was set to 800 ms to ensure 3T_1_ delay between successive pulses, and it was repeated 4 times to achieve satisfactory image quality. We acquired time domain data into 512 × 512 matrices, processed off-line using Prospa 3.2 (Magritek) to result in the magnitude images presented in this study. Final in-plane image resolution was < 20 μm^2^. T2* contrast data were acquired using the same experimental protocol. We collected 10 in-plane echoes to reconstruct a full relaxational picture in different anatomical regions of the skin. Only positive echoes were used for T2* analysis. Final images were analyzed exponentially in each voxel using Prospa 3.2, resulting in the maps highlighting different relaxation behaviors in each skin region. The T2* images were converted into histograms and then further analyzed with a build-in Multipeak fitting routine in IgorPro8.2 (Wavemetrics).

### ^23^Na MRS.

We used a TopSpin 3.2 (Bruker) home-written 2-dimensional TQ-TPPI sequence (23, 25) to determine the fraction of sodium in the bound state in all skin biopsies. Spectral width in the direct dimension was set to 4 KHz, the indirect dimension was incremented using 256 200 μs time increments, recycle delay was 0.1 seconds, and the spectra were collected under 2 minutes. Data were processed with TopSpin3.2, with zero filling data in both dimensions. Line broadening was 5 Hz in doth dimensions, with spectra manually phased in both dimensions. We derived fractions of bound and free sodium by analyzing central traces extracted at zero frequency in the direct dimension, using a build-in Multipeak fitting routine in IgorPro8.2. We used normalized intensities of Lorentzian distributions (32) obtained during spectral deconvolution as fractions of bound and free sodium.

### ^23^Na MRI.

We performed localization of free sodium with TopSpin 3.2 (Bruker) using a home-written nonselective GE MRI protocol. The width of the π/2 pulse was 54 μs, the recycle delay was 0.2 seconds, and the spectral width was 25 kHz. We collected images into 64 × 64 matrices with the phase-encoding time of 1.2 ms and maximum phase and read gradients set to 5% and 6%, respectively, resulting in a field of view (FOV) of 32 × 25 mm^2^. We accumulated 512 transients (image collection within 2 hours). We reconstructed images in Prospa 3.2 (Magritek) by zero filling to 256 × 256 matrices; sinc-squared apodization was performed in both dimensions with consecutive magnitude 2-dimensional Fourier transformation. The resolution of the processed images was approximately 0.125 × 0.098 mm^2^ in the axial plane.

We performed visualization of bound sodium using a TopSpin 3.2 (Bruker) home-written code, with application of TQF before localization of bound sodium with the GE. We set the evolution time to that determined in TQF ^23^Na MRS, by identifying the appropriate evolution time τ where the TQ signal was at maximum. The width of the π/2 pulse was 49 μs, with the width of π pulse in the TQF set to 98 μs, the TQF evolution time τ was set to 3 ms, the recycle delay was 0.2 seconds, and the spectral width was 50 kHz. We collected axial images into 128 × 32 matrices with the phase encoding time of 1.2 ms — with maximum phase and read gradients set to 4% and 5%, respectively — resulting in a FOV of 19 × 60 mm^2^. We accumulated 960 transients (image collection within 2 hours). We reconstructed images in Prospa 3.2 by zero filling to 256 × 256 matrices and sinc-squared apodization performed in both dimensions with consecutive magnitude 2-dimensional Fourier transformation. The resolution of the processed images was approximately 0.074 × 0.234 mm^2^ in the axial plane.

### Histocytochemical analysis.

To provide further insight into the colocalization of MRI-based sodium signals with GAG molecules within the skin dermis layer, we performed histocytochemical staining for GAG molecules within one of the skin samples from a control participant. For this, we used the Alcian blue technique for staining acid mucins. Following dewaxing, we placed precut sections of skin in distilled water. We stained each section in Alcian blue solution (pH 2.5) for 15–20 minutes. Following rinsing in distilled water, we then applied a counterstain in 1% neutral red for 1–2 minutes, rinsed well in distilled water, dehydrated, cleared, and mounted each skin section. For the histocytochemistry analysis, blue positivity indicates the presence of acid mucins (including GAG molecules). To provide an indication of colocalization of MRI-based sodium signals with GAG staining, we superimposed the GAG stained images with ^23^Na MRI slides obtained for both free and bound sodium states.

### Statistics.

We performed all statistical analyses using IgorPro8.2. Samples of fractions of free and bound sodium in skin biopsies were extracted from the analysis of 2-dimensional ^23^Na TQ-TPPI and were subjected to the following tests to determine a final statistical approach for the interpretation of data presented in this study within a significance level of α = 0.05. A serial randomness test suggested that the samples were random. The Kolmogorov–Smirnov test rejected a hypothesis of normal distribution in all samples. The *F* test suggested unequal variances in both samples. Therefore, we used a Wilcoxon signed-rank test rather than *t* test for determination of validity of the null hypothesis.

Multi-element analysis by ICPMS was performed using Thermo Fisher iCAP-Q with a Flatopole collision cell (typically charged with helium gas) upstream of the analytical quadrupole to reduce polyatomic interferences. Internal standards are normally introduced to the sample stream via a T-piece and typically include Scandium (Sc; 50 μg/L), Germanium (Ge; 20 μg/L), Rhodium (Rh; 10 μg/L), and Iridium (Ir; 5 μg/L) in the preferred matrix of 2% HNO_3_; similar concentration levels of HCl are also acceptable. We used external calibration standards in the usual range of 0–100 μg/L (ppb). We introduced samples using a covered autosampler (Cetac ASX-520), through either a concentric glass venturi nebulizer (Thermo Fisher Scientific) or a PEEK Burgener Miramist nebulizer. We performed further sample analysis using Qtegra Intelligent Scientific Data Solution Software (Thermo Fisher Scientific). Skin region samples for ICPMS were prepared using small quantities of 3 major skin regions (≤0.4 g), weighed directly in Digitubes using Analytical balance. Subsequently, 8 mL of nitric acid (HNO_3_, trace metal grade) and 2 mL of hydrogen peroxide (H_2_O_2_, metal grade), were added to each solid phase skin sample, which were then left overnight. We heated each sample for 2 hours at 95°C and then left the samples for 10 minutes to cool down. Subsequently, we added Milli-Q (MilliporeSigma) water to bring the final volume in each digested sample to 50 mL. We also added Milli-Q water directly to the harvested plasma samples (derived from the vacuum packs used to transport each skin sample so no digestion was required), to make a volume of 10 mL. Outcomes of sodium levels in the various skin regions are presented in Table 2.

### Study approval.

We obtained the skin samples from the Arden tissue bank, located at UHCW, with patient consent obtained prior to lower limb amputation or abdominal skin reduction surgery. We obtained ethical approval from West Midlands Research Ethics Committee, United Kingdom.

## Author contributions

GEP and TMB cowrote the paper. GEP designed the ^23^Na MRS and MRI experiments and collected data. CJP collected experimental data and performed ICPMS analysis. PH coordinated the collection and transfer of vacuum-packed skin samples. SJ performed the histocytochemical analysis. PH, CJP, TM, HSR, SJ, and JPO contributed toward the writing of the manuscript. TMB, PH, HSR, JPO, and GEP contributed toward the conception and overall design of the study protocol.

## Supplementary Material

Supplemental data

## Figures and Tables

**Table 1:**
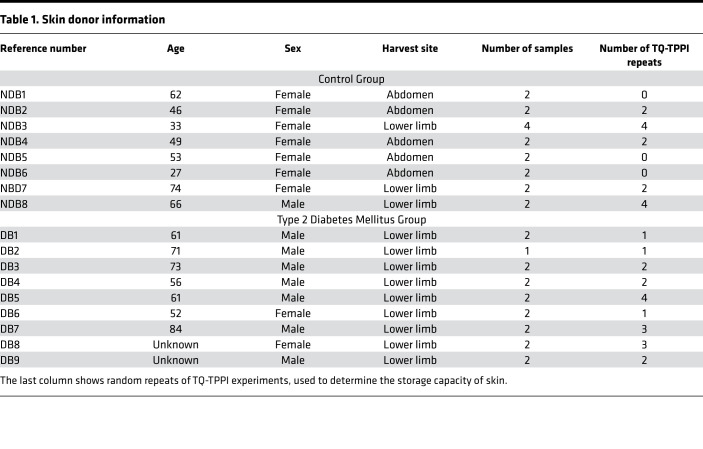
Skin donor information

**Table 2:**
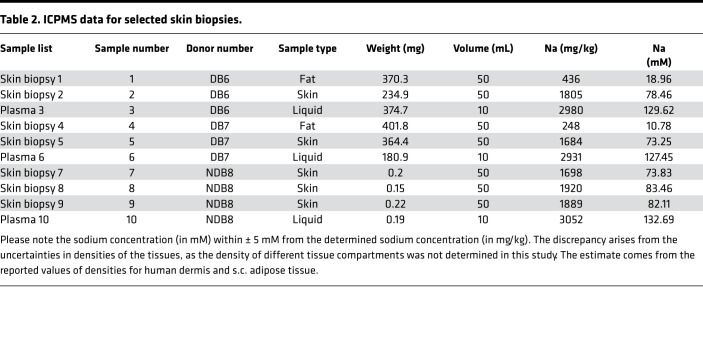
ICPMS data for selected skin biopsies.

## Version 1 05/18/2021

In-Press Preview

## Version 2 06/22/2021

Electronic publication
