# Supplementary material for: Sodium in the dermis colocates to glycosaminoglycan scaffold, with diminishment in type 2 diabetes mellitus
Source: JCI Insight. 2021 Jun 22;6(12):e145470. doi: 10.1172/jci.insight.145470 (PMC8262470; doi:10.1172/jci.insight.145470)
Supplement: Supplemental data [file jciinsight-6-145470-s168.pdf]

Sodium in the Dermis Co-locates to Glycosaminoglycan Scaffold, with Diminishment in Type 2 Diabetes Mellitus

Petra Hanson, Christopher J Philp, Harpal S Randeva, Sean James, J Paul O'Hare, Thomas Meersmann, Galina E Pavlovskaya and Thomas M Barber

Supporting Information

To produce images suitable for co-registration with sodium, we added individual 100 μm ^{1}H MRI slices to create non-slice selective images for better comparisons with sodium imaging. The corresponding proton images are displayed in *Figure 1SI* (a,b) for both control and diabetic skin biopsies. To further discern the anatomy of the skin biopsies, we also show one 200 μm T2 weighted spin-echo slice in *Figure 1SI* (a) where dermis appears as a darker outer layer in the control skin biopsy.

For the purpose of comparison, sodium imaging was performed using the same geometry as used in the ^{1}H MRI scans, and the resultant proton and sodium images are displayed for the diabetic (DB3) skin biopsy in *Figure 1SI* (a) and (b), respectively. Due to the nuclear spin S= 3/2 properties of ^{23}Na, bound sodium could be localised through TQF ^{23}Na MRI, and the corresponding images are shown in *Figure 1SI* (b,d), with the concepts and details explained in the Introduction and Methods sections. As observed from *Figure 1SI* (a), bound sodium in the control skin biopsies located mostly within the dermis layer of the skin, with some deposition of bound sodium also within the connective tissue of the contiguous subcutaneous layer of the skin. We observed a similar pattern for bound sodium within the diabetic skin biopsies, as shown in *Figure 1SI* (d) (coronal series). However, the relative sodium signal-to-noise ratios (SNRs) of bound sodium compared to the SNRs of the respective free sodium were significantly lower in diabetic than in those for control skin biopsies.

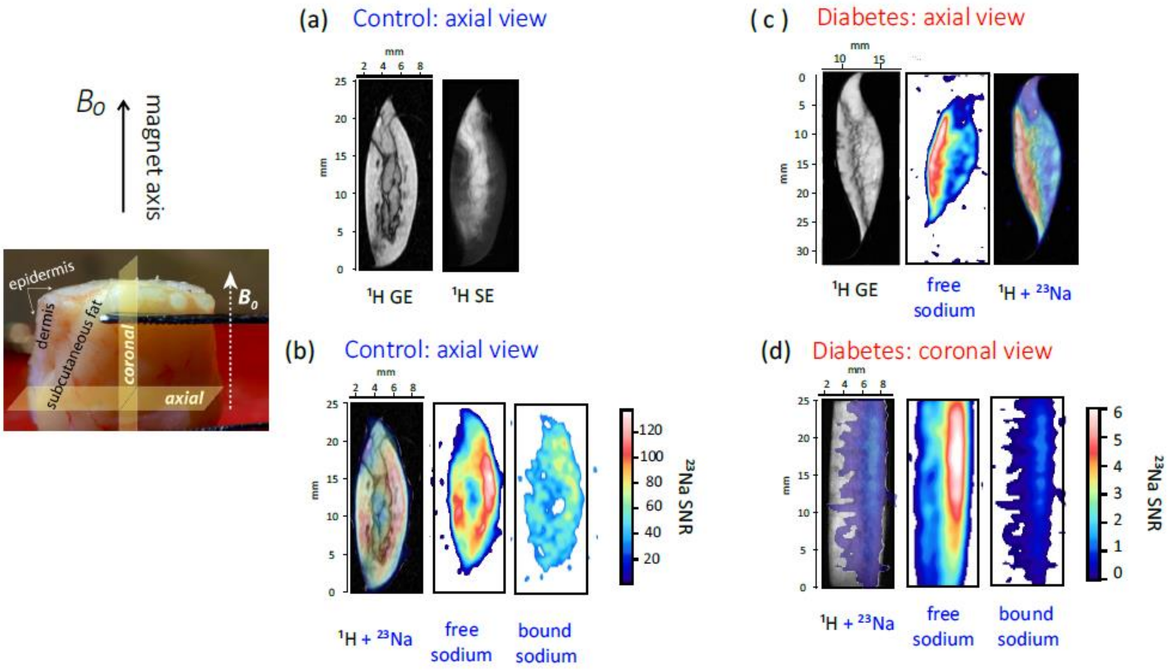

Figure SI1. High-resolution proton and sodium images of skin biopsies. Photograph of a typical skin biopsy with illustration of the orientation of axial and coronal view planes with the respect to the magnetic field direction (ie. MRI magnet axis) is shown in the left panel. (a) ^{1}H high-resolution axial plane MRI of control (NDB8) skin biopsy using gradient echo MRI protocol with TE = 2ms (^{1}H GE). Note that the specimen was folded by the vacuum packing procedure, as identifiable by the enhanced segmentation of the dermis layer obtained with spin-echo (SE) MRI using the same as in ^{1}H GE geometry (dermis appears dark in ^{1}H SE). (b) – Free sodium image of control biopsy collected without additional re-positioning and the corresponding bound sodium image collected with TQF ^{23}Na MRI protocol as described in Introduction and Methods. The ^{23}Na/^{1}H fusion image shows that, in controls, free and bound sodium is localised mostly in the dermis layer. (c) - ^{1}H gradient echo (^{1}H GE) of the diabetic skin biopsy DB3 in the axial plane with corresponding free sodium ^{23}Na MRI and co-registration image. (d) - Coronal projection also demonstrates localisation of the free and bound sodium for DB3 skin biopsy. There is co-registration of the bound sodium spaces with the dermis compartment. Sodium levels expressed in SNR, as explained in the text.

Optimisation of ^{23}Na triple quantum filter. We used a TopSpin 3.2 (Bruker, Germany) home-written ^{23}Na MRS TQF protocol to characterise time evolution of bound sodium in the skin. We used twenty three TQ evolution delays τ to determine a delay where the TQF was at the maximum. We collected 960 transients for each τ delay, with a recycle delay of 0.2 s, thus bringing the total time for the entire series to within 2h. The resultant time series are displayed in Figure 2SI, demonstrating that the maximum of the triple quantum signal occurred at $\tau = 1.5\ ms$. This optimised value of τ was used in the TQ filter added to all imaging protocols for bound sodium visualisation.

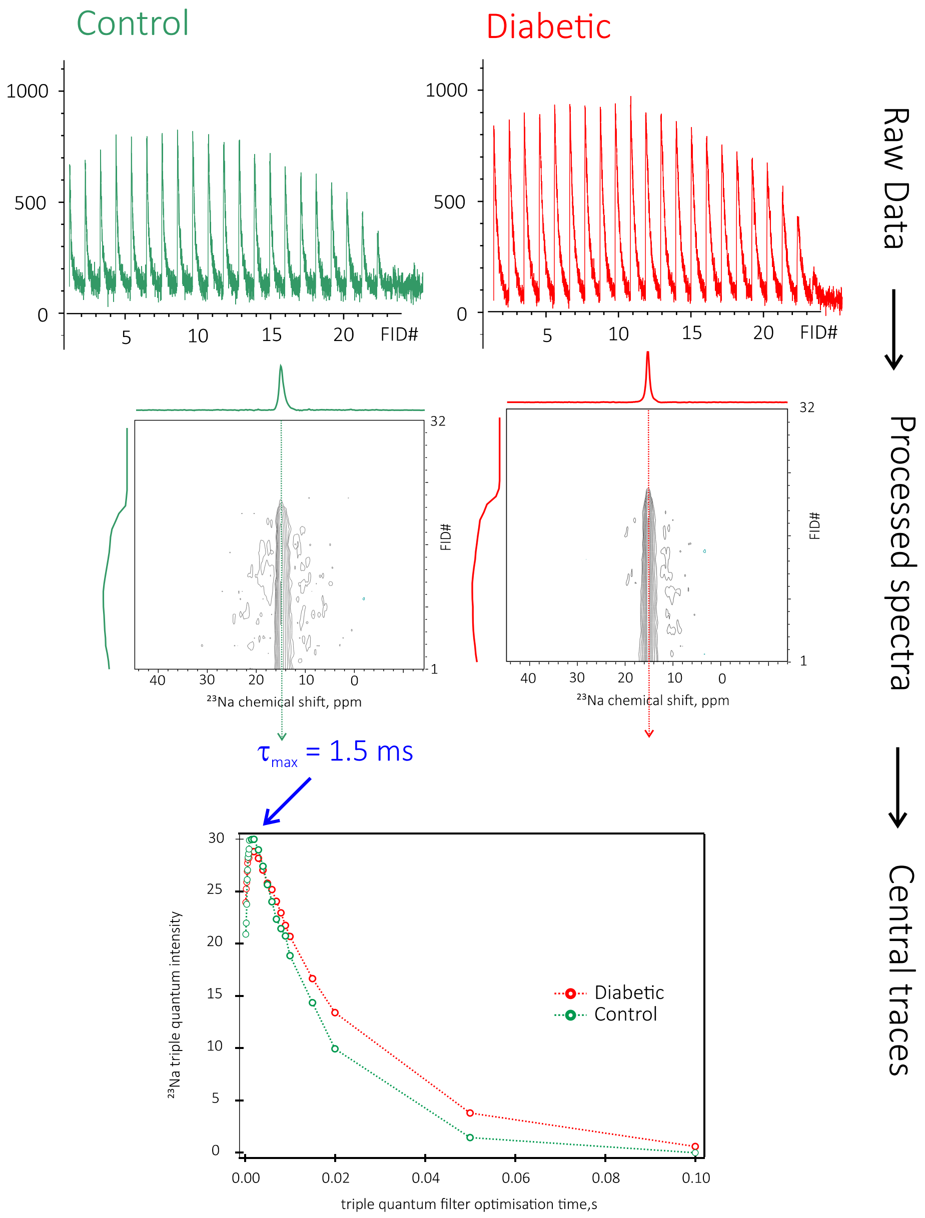

Figure SI2. Optimisation of the triple quantum filter for all imaging TQF experiments. Top row displays raw data for both control and diabetic biopsies, middle row shows processed data, and the bottom raw displays sodium intensities of the central trace as a function of triple quantum filter optimisation time. The maximum of signals for both control and diabetic biopsies appears at $\tau = 1.5\ ms$. Right column shows acquisition an processing pipeline.

^{23}Na MRI saline standards calibration: We scanned a set of NMR tubes filled with 10, 2, 50, 75 and 100 mM of aqueous NaCl solutions with the same parameters as used for sodium image collection from skin biopsies. We converted sodium image intensity to bound and free sodium concentrations using a calibration curve derived the dependence of integrated 23Na intensity in each tube vs known saline concentration in each tube as shown in the graph to the right.

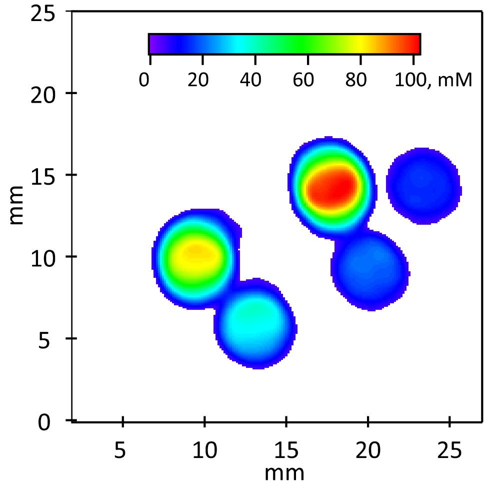

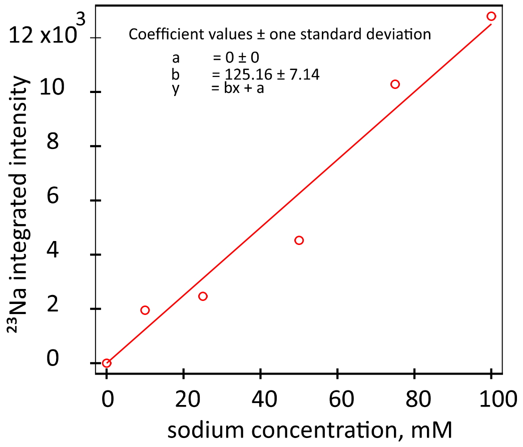

The calibration line was determined for each sodium image independently as additional scaling of ^{23}Na-integrated intensity, obtained from the phantoms to account for differences in the length between the phantoms and of the scanned skin biopsy. We used coronal images for this purpose. Using this methodology, stored (bound) and free sodium levels were determined within ±7 mM for studied skin biopsies.
